# Progress, or Something New

**Published:** 1877-05

**Authors:** 


### Editorial.

### Progress--or, Something New.

In looking over the periodical literature of the last months, we think we may safely say there is manifest desire to propose some new plan or new remedy, something recent, and all the better if wonderful—something that will change established practices or do away with them altogether.

This is presented to the profession under the general head of *Progress.* The activity and restlessness of working thinking minds is shown in desire to change; we change from love of it in some cases, while we all seek earnestly for something better, more certain, more active, more efficient and above all something *more true.* If we patiently study the teachings of the Fathers, and with care and humility improve and correct them rather than ignore and reject them, possibly we may proceed in the safe line of progress; for it must not be doubted that we are making progress in our art, at times with such rapid strides that we are liable to grow dizzy and possibly lose balance. Our new remedies need more time to try them; our new theories and practices have not stood the test of centuries; and our recently improved methods yet require the confirming and regulating processes of time. It is true, we are now ready to see our most cherished practices condemned and rejected—glad to see this if unworthy and defective; but in rejecting them we desire a substitute of that which is safer, truer and better.

In Medicine, what change is being made? Our doctors of forty years practice who have formerly known the whole list of Materia Medica, and during life tested for themselves the virtues of every article, would now hear the list in good regular English, and not recognize the subject under discussion—would infer from the names of remedies, that the Chocktaw tongue was required in the nomenclature of the Materia Medica, to say nothing of the names of diseases. One day last week an educated, highly respected and experienced physician entered our office with his usual rapid steps and earnest manner, at once asking, "What do you know about some of the new remedies I see advertised, such as 'Kava-Kava,' 'Grindelin Squarrosa,' or 'Viburnum Prunifolium,' the last said to be for the prevention of abortion? Do doctors take Viburnum Prunifolium to prevent them from producing abortion, or is it given the women to prevent abortion being produced? What on earth are we all coming to, if our old and established remedies are to give place to this Babel of medical nonsense—this moonshine or sundew of Materia Medica? When, in the name of wonder, will the new United States Pharmacopæia be out? I have lost all recollection of the names of remedial substances, and I come to you to borrow the last work on Materia Medica; for, to tell the truth, I have practiced physic for near half a century, and I never heard these remedies mentioned; and from recent accounts, there must be a power in them never yet appreciated."

To turn his attention we inquired if he had yet learned to reduce hernia by aspiration. "O, yes; I think that is the way my old preceptor used to do it. When all other means failed, he used to bring the legs of the patient up over his shoulders from behind, and rising from the bed suspend them head downwards on his back, sometimes injecting water into the bowel, and he used to say the bowel would go back by gravitation. Now they may call it 'as(s)piration,' for the sake of something new, but I guess it is the same old plan."

